# Phycochemical and Biological Activities From Different Extracts of *Padina antillarum* (Kützing) Piccone

**DOI:** 10.3389/fpls.2022.929368

**Published:** 2022-07-22

**Authors:** Juveria Samar, Ghazala Yasmeen Butt, Anis Ali Shah, Adnan Noor Shah, Sajad Ali, Basit Latief Jan, Nader R. Abdelsalam, Muhammad Hussaan

**Affiliations:** ^1^Department of Botany, Government College University, Lahore, Pakistan; ^2^Department of Botany, The University of Punjab, Lahore, Pakistan; ^3^Department of Botany, Division of Science and Technology, University of Education, Lahore, Pakistan; ^4^Department of Agricultural Engineering, Khwaja Fareed University of Engineering and Information Technology, Rahim Yar Khan, Pakistan; ^5^Department of Biotechnology, Yeungnam University, Gyeongsan, South Korea; ^6^Department of Clinical Pharmacy, College of Pharmacy, King Saud University, Riyadh, Saudi Arabia; ^7^Department of Agricultural Botany, Faculty of Agriculture (Saba Basha), Alexandria University, Alexandria, Egypt; ^8^Department of Botany, Government College University Faisalabad, Faisalabad, Pakistan

**Keywords:** antioxidant, antimicrobial, anthelmintic, antidiabetic, *Padina antillarum*

## Abstract

Seaweeds are non-vascular, photosynthetic that inhabit the coastal regions commonly within rocky intertidal or submerged reef-like habitats and have been one of the richest and most promising sources of bioactive primary and secondary metabolites with antimicrobial properties. They selectively absorb elements like Na, K, Ca, Mg, I, and Br from the seawater and accumulate them in their thalli. *Padina antillarum* (Kützing) Piccone is a member of Phaeophycota and has remarkable phycochemistry as well as bioactivity. The phycochemical tests of the different extracts showed the presence of alkaloids, terpenoids, saponins, tannins, steroids, and phenols. The relative percentage of Oxirane, tetradecyl (C_16_H_32_O), and Cyclononasiloxane (C_18_H_54_O_9_Si_9_) are higher while Tetrasiloxane (C_16_H_50_O_7_Si_8_) is lowest in Gas Chromatography – Mass Spectrometry analysis. FRAP, %inhibition, the total antioxidant value of *P. antillarum* was higher in methanolic extract. Hexane, chloroform extracts showed no zone of inhibition against *Bacillus subtilis, Escherichia coli, Klebsiella pneumonia, Staphylococcus aureus*, and *Staphylococcus epidermidis.* The methanolic extract of *P. antillarum* exhibits a maximum zone of inhibition against *S. epidermidis* (18.66 ± 0.09). Antifungal activity of the *P. antillarum* in hexane extract exhibited no zone of inhibition against *Aspergillus niger* and *Penicillium notatum* while the chloroform extract yields maximum zone (37 ± 0.012, 21.66 ± 0.03). Diabetes mellitus is one of the most familiar chronic diseases associated with carbohydrate metabolism. It is also an indication of co-morbidities such as obesity, hypertension, and hyperlipidemia which are metabolic complications of both clinical and experimental diabetes. The treatment of *P. antillarum* methanol extract in mice reduced the body weight loss, low level of triglycerides, and elevated HDL cholesterol level as compared to diabetic mice.

## Introduction

The Karachi Coast (100 km) touches the Arabian Sea. It includes beaches and numerous islands. The coastal waters around Manora, Sandspit, Hawkesbay, Buleji, Paradise Point, Pacha, Nathiagali, and Cape Monze are inhabited by a variety of marine benthic algae (Shameel and Tanaka, 1992). Although a lot of work has been done on their taxonomy and distribution, as well as morpho-ecological and phycochemistry studies (Hameed et al., 2000, 2001; Shameel et al., 2000), little data are available in the literature related to their bioactivity and elemental composition (Rizvi and Shameel, 2005). A total of 234 species and 110 genera of seaweeds are reported from the coast of Balochistan, demonstrating a great biological diversity, distributed among 57 families, 33 orders, 12 classes and 6 divisions (Shameel, 2001). Many studies have been made on the standing crop and biomass of seaweeds like Shameel, 1987 served seaweed from the coast of Lasbella, Pakistan. Saleem (1965), Saifullah (1973), and Saifullah (2009) studied the distribution and biomass of marine algae along Karachi. Qari and Qasim (1994) investigated the Seasonal change in the standing crop of intertidal seaweeds from the Buleji and Manora coasts of Karachi. Qari et al. (2014) studied Phytomass on a natural bed of seaweed at Paradise Point, Karachi coast. Qari (2017) assessed the seaweed diversity and distribution at the beach of Nathia gali, Karachi, Pakistan. Seaweeds are non-vascular, photosynthetic that inhabit the coastal regions commonly within rocky intertidal or submerged reef-like habitats and have been one of the richest and most promising sources of bioactive primary and secondary metabolites with antimicrobial properties (Chapman, 1950; Jebasingh et al., 2011). Seaweeds were consumed as whole food since ancient times, and they still have great economic importance. Seaweeds are considered a nutrient-rich food as they are a good source of minerals, vitamins (A, B_1_, B_2_, B_9_, B_12_, C, D, E, and K), essential minerals (calcium, iron, iodine, magnesium, phosphorus, potassium, zinc, copper, manganese, selenium, and fluoride), dietary fibers (Rajapakse and Kim, 2011; Dhargalkar, 2015; Pereira, 2018; Shannon and Abu-Ghannam, 2019), protein, essential amino acids, and polyphenols, which exhibit antioxidant and anti-inflammatory properties (Panzella and Napolitano, 2017). Seaweeds possess a low lipid content, nonetheless enriched in polyunsaturated fatty acids. This characteristic makes them even more attractive, as they are a healthy, nutritive, and low-caloric food (Pereira, 2018). The accumulated elements vary from species to species. Different types of dietary fibers exist depending on the seaweed phyla. For brown seaweeds (Phaeophyta) the soluble fibers are alginates, fucans, and laminarians (Peñalver et al., 2020). A plague of concern is the biological effects of natural antioxidants, encompassed in the attack against oxidative trauma that grounds aging, the release, and progress of numerous diseases such as cancer, cardiovascular accidents, inflammatory diseases, and neurodegenerative diseases (Martemucci et al., 2022). Macroalgae are available, safe, cheap, and due to their bioactive properties, with positive effects on human health have received considerable attention. *Padina* can be utilized as food, fodder, and bio-fertilizer. The brown alga is well known and is utilized for its antimicrobial, insecticidal, antioxidants, antibiotics, anti-inflammatory, hypo-allergenic, hepatoprotective, and antidiabetic activities. The macroalgae like *Padina* play important role in environmental monitoring and management of coastal marine ecosystems. They can be considered biological indicators and can also be utilized in the phytoremediation, for the management of contaminants in coastal marine ecosystems (Ansari et al., 2019). A few species of *Padina* have been used traditionally as a food source in many coastal parts of the world. *Padina* is also reported as a substitute for salts for patients with high blood pressure (Novaczek and Athy, 2001). Seaweed distribution and bioactive compounds from the coastline of Pakistan have already been studied (Yasmeen et al., 2021) and in spite of some scattered studies in this area are not completely known yet. *Padina antillarum* ((Kützing) Piccone was found as a drifted material or benthic in bulk amount at Karachi coast. This type of work is not done on this species in Pakistan. Objectives are following:

(1) Extraction and analysis of phycochemicals from *Padina antillarum*.

(2) Evaluation of antimicrobial, antioxidant, antidiabetic, and anthelmintic potential of *Padina antillarum*.

## Materials and Methods

### Collection of Seaweeds

*Padina antillarum* (Kützing) Piccone was collected from the intertidal and subtidal habitat of the Manora, Hawkesbay and Buleji between longitude 66° 59″ E and latitude 24° 48″ N were the suitable place for the collection of drifted seaweed at the coast of Karachi, Pakistan. Collection was performed during the April 2018 to November 2020. The drifted seaweeds were collected from the rocks by hands, forceps, and scrapers. Collection bags of different sizes and plastic containers were used to keep them preserved. Zipper bags of different sizes were used for preservation. The collected healthy seaweeds were brought to the laboratory for cleaning and washed thoroughly with tap water with an adequate amount of salt. The washed algal samples were dried under shade on bloating paper and made some herbarium. The herbarium of collected algal species is kept in Kashyap Botany Museum, GC University, Lahore, Pakistan. Seaweeds were identified by using standard literature and verified by the Department of Botany, University of Karachi, Karachi. Systematic positions of these algal species were followed according to the classification of Shameel and Tanaka (1992) and Shameel (2001, 2008). Powder algal material soaking was performed based on the standard static state maceration technique using *n* hexane, chloroform, and methanol solvents.

#### Morphological Analysis

The morphological analysis includes the appearance of the *P. antillarum* according to Shameel (2008) and Abbas (2014).

### Analysis of the Biochemical Composition

#### Moisture Content

Moisture content algal samples were determined according to the standard procedure of Association of Official Analytical Chemists [AOAC] (1997). For this purpose, porcelain crucibles were dried and placed in an oven for 3 h at 105°C, and then allowed to cool. After cooling, crucibles were weighed and 1 g each algal powdered material of the sample was placed in the crucible and placed in an oven at 105°C for 3 h. Crucibles were then allowed to cool and weighed afterward.

#### Ash Content

Ash content was determined according to the standard protocol of Association of Official Analytical Chemists [AOAC] (1997). For the process, crucibles were placed in a furnace at 550°C overnight in order to get rid of any impurities if present. Crucibles were then allowed to cool in a desiccator after cooling crucibles were weighed up to 3 decimal points. About 1 g of the equally weighed powdered algal material sample under examination was then placed in crucibles and heated at 550°C in a furnace overnight. Crucibles along with ash were then weighed after cooling.

#### Porosity Test

The porosity test examined dry weight. The material was inserted into the water and examined the mass. The porosity value could be known by comparing the dry weight material and the wet material mass. The procedure was done by mixing 0.3 g of material with 150 _μ L_ of water, after setting measured the dry weight sample material was dipped, into 6 ml water and measured the wet sample weight. The dry and wet weight difference was calculated (Widiyanti and Siswanto, 2012).

#### Qualitative Analysis

The qualitative determination of extracts was carried out according to Harbone (1973) and Ayoola et al. (2008). These are as follows Glycosides, cardic glycosides (Keller Killani test) anthroquinones (Chloroform, Borntager test), alkaloids (Dragendroff reagent, Mayer’s reagent, Wanger test), flavonoids (Sodium hydroxide test, Ammonia test, Ferric chloride test), saponins (Frothing test), terpenoids (Salkowski Test), phenols (Ferric Chloride Test), amino acids (Ninhydrin Test), proteins (Biuret Test), steroids, coumarins, beta cyanins, reducing sugar (Fehling Test, Iodine Test, Tollen’s Reagent), oils and resins, phlobatanins, quinones, and tannins (Ferric Chloride Test, Match test).

##### Total Alkaloid and Fat Contents

A sample of 3 g was taken in a beaker, and 200 ml of 20% ethanolic acetic acid was added. The beaker was covered and left for 4 h. The extract was filtered and volume was reduced to one-quarter of the initial, using a water bath. Then concentrated NH_4_OH was added slowly dropwise to the extract until the precipitation get complete. The precipitates were left for settling then filtered, dried, and weighed (Poornima and Ravishankar, 2009). Total fat was extracted by Soxhlet apparatus 10 g sample was taken into a thimble. The empty extraction flask was weighed. And 250 ml of *n*-hexane was used as an extraction solvent. The refluxing was continued for 8 h at the boiling point of *n*-hexane. After extraction, the solvent was evaporated from the flask and weighed the flask with extracted crude oil. The percentage of the extracted oil was calculated by using the equation (Association of Official Analytical Chemists [AOAC], 1997).


$$\%\mathrm{of}\mathrm{oil}=\frac{\mathrm{Extracted}\,\mathrm{oil}}{\mathrm{Dry}\,\mathrm{sample}}\times 100$$

#### Nutritive Analysis

##### Protein Estimation (Biuret Method)

The protein content was estimated by the Biuret method (Raymont et al., 1964). Incubate the mixture prepared, using 5 mg of dried powdered sample, 1 ml of distilled water followed by addition of 4 ml biuret reagent, for 30 min at room temperature. Then the mixture was centrifuged for 10 min at 4,000 rpm. The supernatant was collected and the optical density was measured in a Spectrophotometer at 540 nm. The protein content was calculated using Bovine Serum Albumin as standard and expressed as ^mg^/_g_ protein.

##### Carbohydrates Estimation

The carbohydrate content was estimated by the Dubois method (Dubois et al., 1956). Add 20 mg of dried seaweed powder and 1 ml of 4% phenol solution and 5 ml of concentrated sulfuric acid. After that, they were kept in a dark room for 30 min. The color intensity developed was read in a spectrophotometer at 490 nm. Sugar content was calculated by referring to a standard D-Glucose and the results have were expressed as ^mg^/_g_ sugar.

##### Lipids Estimation

The lipid content was estimated using chloroform–methanol mixture as described by Folch et al. (1957). Add 400 mg of sample to 5 ml chloroform–methanol (2:1) mixture. The mixture was incubated at room temperature for 24 h. After incubation, the mixture was filtered using filter paper. The filtrate was collected in a 10 ml pre-weighed beaker. The chloroform–methanol mixture was evaporated on a hot plate leaving a residue at the bottom of the beaker. The beaker with the residue and the weight of the empty beaker was calculated to know the weight of the lipid present in the sample.

### Elemental Analysis

Oven dried samples were accurately weighed in 0.2 g quantities in a dry conical flask and 10 ml of diacid mixture (2:5 of nitric and perchloric acid) were added. The contents of the conical flask were allowed to stand for a few hours for cold digestion. The mixture was then kept on a hot plate, and the contents were digested by increasing the temperature. The digestion continued until the content became colorless. The digestion material was filtered through Whatmann No. 40 filter paper, and the filtrate collected was diluted to a suitable volume and fed into an ICP–Perkin Elmer Mayer Optical Emission Spectrophotometer (Association of Official Analytical Chemists [AOAC], 1995).

#### Experiments to Characterize Compounds

##### Fourier Transform Infrared Analysis

Fourier Transform Infrared Analysis (FTIR) analysis was performed using Perk in Elmer Spectrophotometer system, which was used to detect the characteristics of peaks and their functional groups. The peak values of the FTIR were recorded. Each and every analysis was repeated twice and confirmed the spectrum (Janakiraman et al., 2011).

##### Gas Chromatography – Mass Spectrometry Analysis

Column chromatography is one of the most useful methods for the separation and purification of crude extract. The methanolic crude extracts were applied in a silica gel column (230–400 mech) packed with chloroform and eluted with a mixture of chloroform and methanol after purification, the fractions were stored at a temperature of 20°C. The compound separated from the column was analyzed using Gas Chromatography – Mass Spectrometry (GC-MS). Chemical compounds in the sample were analyzed using Shimadzu GC-MSQP-2010A. Helium (He) gas was used in it. The flow rate of He gas was 32 ml/min. The column temperature was about 70–250°C with an increasing rate of 8°C per minute.

#### Biological Activities

##### Antioxidant

###### 2,2′Azino-Bis (3-Ethylbenzothia Zoline-*b*-Sulfuric Acid) Assay

ABTS^++^ assay was performed as described by Re et al. (1999) for the estimation of antioxidant potential. It was produced by reacting ABTS^++^ aqueous solution with 2.45 mM potassium persulfate at room temperature for 16 h. The ABTS^++^ solution was diluted with Phosphate buffered saline (PBS), pH 7.0 to an absorbance of 0.70 at 734 nm. Afterward, 2.9 ml of this solution (with adjusted and recorded reading) was dispensed in a test tube followed by the addition of 10 μl of algal extracts. After precisely noting at an interval of 8 min, absorbance was measured at 734 nm. A dose response curve of Trolox was organized by plotting its absorbance at 734 nm.

###### 2,2–Diphenyl-1-Picrylhydrazyl Radical Scavenging Assay

DPPH radical scavenging activity was evaluated according to the method of Brand-Williams et al. (1995). Prepare four dilutions of each algal extract. Each DPPH reaction test tube was prepared by adding 1 ml of algal extract and 3 ml of freshly prepared DPPH solution. The test tubes were then incubated for 45–60 min in dark at room temperature. During the incubation period, reduction of the DPPH mixture occurs which is evident by the change in color from purple to yellow. The absorbance of samples was recorded at 517 nm by spectrophotometer. Ascorbic acid was used as a standard whereas methanol was used as a blank. The remaining DPPH radical percentage was calculated.


$$\%\mathrm{DPPH}=\frac{A\,\text{sample}-A\,\text{control}}{A\,\text{control}}\times 100$$

###### Ferric Reducing Anti-oxidant Power Evaluation

Ferric reducing the antioxidant ability of different algal extracts was evaluated according to the protocol of Benzie and Strain (1996). For this evaluation, 1 ml of each seaweed extract was treated with a 3 ml working solution of FRAP reagent followed by keeping it under 30 min dark period. The absorbance was chronicled at 593 nm. Standard Trolox was recorded as micromoles of Trolox equivalent (TE) per ml which were evaluated by a standard curve-derived equation.

###### Ferric Thiocyanate Investigation

FTC was predicted by the scheme developed by Valento et al. (2002). The investigation solution was formulated by the supplementation of 0.1 ml (500 μg/ml) seaweed macerate into 2 ml phosphate buffer and 2.5 ml linoleic and incubated for 24 h at 40°C. After the accomplishment of incubation period, 0.1 ml of stemmed mixture was dissolved into 0.1 ml (20 mM) FeCl_2_, 0.1 mL of (30%) ammonium thiocyanate and 5 ml of (75%) ethanol. The acquired mixtures were retained for 3 min at room temperature and checked the optical density at 500 nm, while butylated hydroxyl toluene (BHT) was affianced as standard. The percentage of inhibition is calculated using the following formula:


$$\%\mathrm{Inhibition}=\left[\left(\frac{1-\mathrm{Sample}\,\mathrm{Absorbance}}{\mathrm{Control}\,\mathrm{Absorbance}}\right)\times 100\right]$$

###### Metal Chelating Activity

Dinis et al. (1994) guidelines for metal chelating activity were espoused for estimating the seaweed extracts. Prepare mixture using 1 ml of seaweed macerated extract, add 50 μl of 2 mM FeCl_2_ and 0.2 ml of 5 mM ferrozine solution. This mixture was vigorously shaken followed by incubation at room temperature for 10 min. Absorbance was measured at 562 nm. Percentage inhibition of ferrozine-Fe^2+^ complex development was estimated as:


$$\%\mathrm{inhibition}=\frac{\text{Absorbance}\,\text{control}-\mathrm{Absorbance}\,\mathrm{sample}}{\mathrm{Absorbance}\,\mathrm{control}}\times 100$$

###### Total Anti-oxidant Activity

Total antioxidant potential of algal extracts was determined by phosphomolybdenum method of Prieto et al. (1999). For each test tube of 1 ml seaweed extract add 4 ml of reagent. Test tubes were incubated at 95°C for 80–90 min in a water bath and cool them in a desiccator at room temperature. The absorbance of samples is recorded at a 695 nm UV spectrophotometer. Ascorbic acid was used as a standard and results were documented as AA μg/ml.

###### Total Flavonoid Content

Aluminum chloride colorimetric techniques were evaluated on different extracts of *P. antillarum* samples for determination of the total flavonoid contents according to Zhishen et al. (1999). About 1 ml of each seaweed extract was taken in a test tube, 0.2 ml of distilled water was added to each tube, by further addition of 0.15 ml of 5% NaNO_2_ solution and this mixture was then incubated for 5 min at room temperature. After incubation 0.15 ml of 10%, AlCl_3_ solution was added to the mixture and the solution was allowed to stand at room temperature for 6 min. Then 2 ml of 4% NaOH was added to the mixture. The final volume of this mixture in the test tube was made 5 ml with distilled water, the mixture was shaken well and left to stand at room temperature for 15 min. Absorbance was measured at 510 nm and total flavonoid content was evaluated as mg equivalent of Rutin (mg Ru/g).

###### Total Phenolic Content

Total phenolic content was estimated following the procedure of Makkar et al. (1993). For this determination quantified amounts of Folin-Ciocalteu (FC) reagent were commercially prepared and sodium carbonate was taken into account. Take 1 ml of each seaweed extract and add 2.8 ml of 10% Na_2_CO_3_ and 0.1 ml of 2N FC reagent. This mixture was then incubated at 25°C for 40 min. After incubation, the absorbance was recorded at 725 nm. Gallic acid was taken as standard and results were documented as GAE ^μ^*^g^*/_ml_.

###### Total Carotenoid Content

Total carotenoid contents were estimated by using Hess et al. (1991) protocol. In the test tube, 300 μl (500 ^μ^*^g^*/_ml_) algal extract was placed in 1.2 ml *n*-hexane and 300 μl deionized water, followed by centrifugation at 2000 rpm for 5 min. Ultimately, the supernatant was separated and its absorbance was taken at 35 nm using UV-vis spectrophotometer. Results were represented in terms of reference curve of β -carotene as mg β -carotene/100 g dried sample (mgBC/100 g).

##### Antimicrobial Activity

Antibacterial activity of different extracts was performed according to Jorgensen et al. (2007). About 3 g positive bacteria (*Staphylococcus aureus* (ATCC 14923), *Bacillus subtilis* (ATCC 15029) *S. epidermidis* (ATCC14990)) and 2 g negative bacteria (*Klebsiella pneumonia* (ATCC700721) *Escherichia coli* (ATCC 14962) while two fungal strains (*Aspergillus niger* and *Penicillium notatum)* were used. For the bacterial strains sterilized Nutrient Agar and for fungal strains, Potato dextrose agar was used as media. The bacterial cultures used for determining the antibacterial activity were spread on the Nutrient Agar medium, formulated under the directions given by American Public Health Association [APHA] (1948) following Cruickshank et al. (1975) protocol. As per Mc- Farland turbidity standard 1.5 × 10^8^ CFU/ml was the maintained for inoculum.

###### Agar Well Diffusion Method

Agar well diffusion method was formulated by Kirby et al. (1957) for estimation of antimicrobial activity. The procedure was later modified as per Jorgensen et al. (2007). Crude seaweed extract inhibits bacterial growth and forms a zone. The inoculum was homogeneously spread on the solidified medium. A well was made at the center of the Petri plate by 0.1–0.3 Cork borer followed by injected the algal material into the well. Petri plates were incubated at 27 ± 2°C for 48 h for fungal activity and at 37 ± 2°C for bacterial growth. The entire experiment was run in triplicates for accurate results.

###### Measurement of Zone of Inhibition

The zone of inhibition was scanned using Agar well diffusion method. Antibiotics disk taken as positive control while *n*-hexane, chloroform, and methanol were used as negative control. Once the incubation period was completed the zone of inhibition molded by the algal extract was measured in mm. The area with the apparent stroke of the algal extract was recorded as a zone; no progress was discernable as 0 mm zone. Sometimes, the zone shaped by the algal extract isn’t symmetrical. In such situations zone is measured from center to side from different angles.

###### Estimation of Minimum Inhibitory Concentration

The determination of MIC was accessed by the broth dilution method (Jorgensen et al., 2007). Different concentrations of 0.625–10 mg/ml of the different extracts were made. About 20 μl of growing inoculum of tested bacteria/fungal species adjusted to 10^7^ CFU/ml, was added to each well and incubated for 24 h at 29 and 37°C. The lowest sample concentration showing no visible antimicrobial growth was considered as the MIC value expressed in mg/ml.

##### Anthelmintic Investigation

According to Selvi and Santhanam (2016), the seaweed extract was evaluated for anthelmintic inquiry. The contemplation on the anthelmintic activity of the seaweed extracts was piloted against the gastro-intestinal nematode *Haemonchus contortus* at the desired concentrations (10, 20, 50, and 100 mg/ml). The tested worms were considered as six groups for evaluation of the anthelmintic potential of seaweed extracts. Seaweed extract of 10 ml (saline solution used as control and Piperazine citrate as standard) was imperiled to each plate monitored by the addition of 5 uniform-sized nematodes in it. The paralysis to the death time period was noted in minutes.

##### Antidiabetic Activity

According to Radhika and Priya (2015) the activity was divided into two phases, i.e., toxicity test and hyperglycemic activity. Ethical conditions regarding the use of laboratory animals were considered before the start of the experiment. The institutional bioethical committee of Government College University approved ethical conditions (No. GCU-IIB-333) to be as per standards.

###### Animal Model

Healthy swiss albino male mice (22–27 g) approximately 6–7 weeks old were purchased from The University of Lahore animal house. Before the commencement of the experiment animals were acclimatized (Hong et al., 2011). Animal house temperature was maintained between 19 and 25°C and humidity 30–37%. The facility was provided with 12-h light and dark. Standard polypropylene mice cages with stainless steel top grill were used. The cages were cleaned with alcohol on regular basis and a clean paddy husk was used as bedding material. Standard mice pellet feed and water *ad libitum* were provided to the mice except for the fasting period during which only water was provided (Banu and Umamageswari, 2011).

###### Toxicity Study

In the experiment, a total of 28 swiss albino mice were used for the administration of seaweed extracts with a test compound at the highest dose of 2,000 mg/Kg. The dose of each extract was administrated orally to the overnight fasted mice. The sign of possible toxicity was observed every 3 h during the first 24 h and every day for 14 days. Individual animal weight was noted down and any sign or symptoms of toxicity, changes in fur, skin, eyes, and mortality were observed for 14 days (Lee et al., 2002; Mohapatra et al., 2016). The groups are as follows Group-I: Vehicle control (distilled water) Group-II: Methanolic extract of *P. antillarum*.

###### Antihyperglycemic Activity

Swiss albino mice (20–30 Kgs) 6–7 weeks old were selected for the antihyperglycemic activity. Before commencement of the experiment the mice were acclimatized to laboratory conditions for 1 week under standard conditions.

###### Induction of Hyperglycemia

Alloxan monohydrate was used as a diabetogenic agent. Alloxan selectively destroys beta cells of the pancreas with irreversible necrosis and it can be generating chronic hyperglycemia. Before the induction of diabetes, the mice were fasted overnight (had free access to water). A freshly prepared dose of 200 mg/Kg Alloxan monohydrate dissolved in 10 mg/Kg body weight distilled water was injected intraperitoneally. Prevention from hypoglycemia the animal is immediately provided with the standard diet. On the fifth day of induction, diabetes was checked by a small incision on the tail for 6 h fasted animal, and blood glucose level was monitored to confirmation of diabetes. Mice with 300 mg/kg blood glucose levels were considered diabetic (Mohapatra et al., 2016).

###### Experimental Design

In the experiment, a total of 36 mice (27 diabetic mice and 9 normal mice in group) were used. The diabetic mice were divided into four groups after the induction of alloxan diabetes (Radhika and Priya, 2015).

###### Assessment of Body Weight

The body weight of each mouse was measured regularly after the induction of diabetes.

**Table:** 

Group-I:	(Treatment group) Diabetic mice received an extract of *Padina* at a dose of 200 mg/kg using intra-gastric tube for 14 days. Dose prepared in CMC.
Group-II:	(Positive control group) Diabetic mice received Glipizide at a dose of 5 mg/kg using intra-gastric tube for 14 days. Dose prepared in distilled water.
Group-III:	(Diabetic control group) Diabetic mice received water *at* a dose of 200 mg/kg using intra-gastric tube for 14 days.
Group-IV:	(Normal control group) non-diabetic mice kept in this group
	

###### Determination of Blood Glucose Level

Blood glucose levels of all experimental groups was measured after the induction of diabetes on the 5th day prior to 6 h fasting. Blood glucose level was also determined on 7th and 14th days by using a standard glucometer (Trinder, 1969).

###### Oral Glucose Test

At the end of 14th days study, an oral glucose tolerance test was performed to monitor the clearance of oral glucose load from the body (Mohapatra et al., 2016). Mice were kept overnight fast before administration of 2 g dextrose/kg body weight. About 2 g of dextrose was injected into the mice by glucose gavage through an oral route. 10 × body weight(g) = 20% dextrose. Blood glucose levels were measured at 0, 30, 60, and 120 min after the administration of glucose. Blood glucose level was checked by a glucometer.

###### Hematological and Biochemical Parameters

Blood samples were assessed for various hematological and biochemical analyses as red blood cell, white blood cell, etc.

#### Statistical Analysis

All the readings were recorded in triplicate and an excel sheet was used to statistically analyze data. All the data were expressed as Mean ± SEM. ANOVA was applied where it was necessary. Differences were considered to be statistically significant if *p* < 0.05.

## Results

### Morphological Description

**FIGURE 1:**
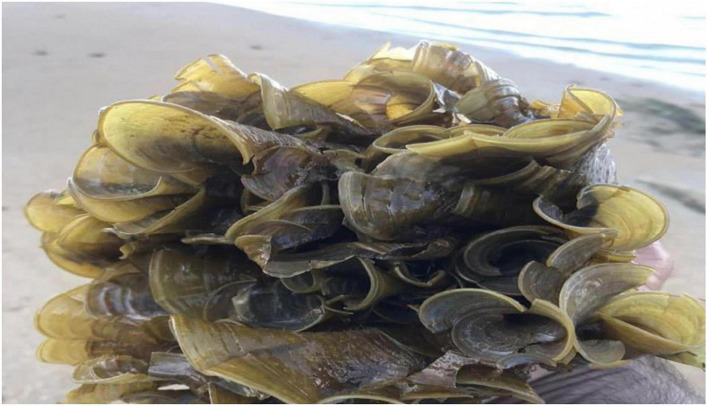
*Padina antillarum* (Kützing) Piccone.

Thalli olive green or dark green in color (Figure 1) irregularly branched; margins smooth or slightly undulate, apex enrolled, surface smooth; sporangia present in double sporangial lines, sporangial lines and hair lines alternate to each other; attached with the help of a small compact (Shameel and Tanaka, 1992) hold fast, 0.5–1.5 cm broad and 0.7–2.0 cm long; thallus divided into many lobes up to ^3^/_4_ part of the thallus, many clefts present on the thallus; thalli 7–15 cm long, 7–12 cm broad at the apex, 10–12 cm broad to the middle and 7.5–10.0 cm broad at the base (Abbas and Shameel, 2011; Abbas, 2014).

### Physical Analysis

The ash content, moisture content, and porosity of *P. antillarum* were 73.8, 5.8, and 5.79 g, respectively.

### Qualitative Analysis

The qualitative phycochemical tests were executed to ascertain the presence of different chemicals in the sample, e.g., glycosides, alkaloids, flavonoids, reducing sugar, tannins, saponins, terpenoids, phenols, amino acids, protein, oil and resins, phlobatanins, quinones, steroids, coumarins and β cyanine. The presence of the different chemicals may vary as in the different extraction solvents for maceration. The qualitative analysis of phycochemistry (Table 1) was assessed according to the standard protocols.

**TABLE 1:** Qualitative analysis of *Padina antillarum.*

Constituents	Phycochemical test	*n*-Hexane	Chloroform	Methanol
Glycosides	Keller-killiani	–	+	+
	Deoxy sugars	–	+	+
Anthroquinone	Chloroform test	–	–	–
	Bornträger’s	–	–	–
Alkaloids	Dragebdroff’s	+	+	–
	Mayer’s	+	+	+
	Wanger’s	+	+	+
Flavonoids	Sodium Hydroxide	+	+	+
	Ammonia	+	+	+
	Ferric Chloride	–	+	+
Reducing Sugar	Fehling	–	+	+
	Iodine	+	+	–
	Tollen’s	–	+	+
Tannins	Ferric chloride	+	+	+
	Match stick	+	+	+
Saponins	Frothing	+	+	+
Terpenoids	Salkowski	+	–	+
Phenols	Ferric chloride	+	+	+
Amino Acids	Ninhydrin	+	+	+
Protein	Biuret	+	+	+
Oil and Resins	Oil	+	+	+
Phlobatanins	Hydrochloride	–	–	–
Quinones	Sulfuric acid	+	+	–
Steroids	Chloroform	+	+	–
Coumarins	Sodium Hydroxide	–	+	+
β Cyanine	Sodiumhydroxide	+	–	+

*+, positive; –, negative.*

### Quantitative Analysis

#### Total Fats and Alkaloids Contents

**FIGURE 2:**
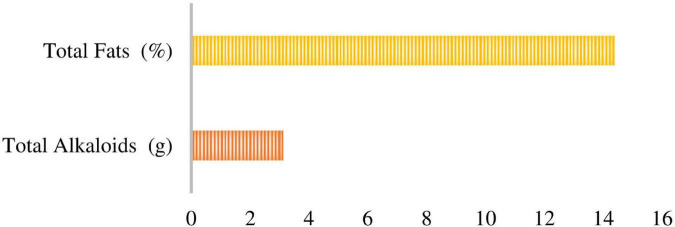
A cluster bar showed the total Fat and Alkaloid contents.

Investigation for total fats in *P. antillarum* was performed by standard Association of Official Analytical Chemists [AOAC] (1997) soxhlet extraction method while the total alkaloid contents were determined according to Poornima and Ravishankar (2009). Most of the algae had fat less than 4% of dry weight but as compared to this several marine algae have high values (Mcdermid and Stuercke, 2003). The total fat contents and total alkaloid of *P. antillarum* are 14.4% and 3.12 g, respectively (Figure 2).

### Nutritive Analysis

The nutritive value of algae can be determined by carbohydrates, protein, and lipids in the sample. The assessment of carbohydrates was expressed in mg/g as referred to as the standard curve of D-glucose. A total of 46.14 mg/g sugar, 107.6 mg/g protein, and 4% lipids contents were estimated.

### Elemental Analysis

*Padina antillarum* has calcium in the highest amount 258.943 while cadmium is present in the lowest amount –0.0279. Other minerals such as potassium 14.972, sodium 17.6543, magnesium 10.7506, iron 4.3142, zinc 0.3940, and lead 0.0151.

### Fourier Transform Infrared Spectroscopy Analysis

**FIGURE 3:**
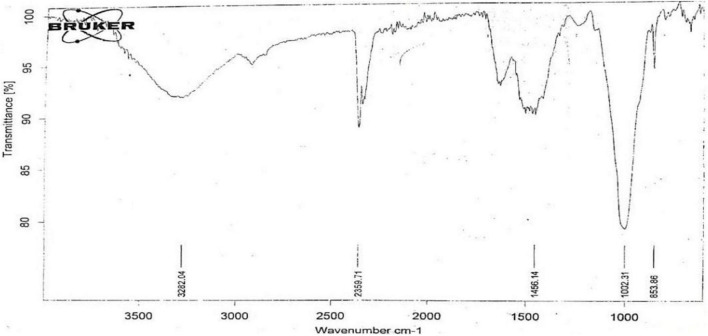
Fourier Transform Infrared Analysis (FTIR) scan of *Padina antillarum* (Kützing) Piccone.

The active components of the functional group in a sample could be identified by the peak value in a region by Fourier transform infrared spectroscopy (FT-IR). The peak ratios separated functional groups of the components as passed the *P. antillarum* crude powder into the FT-IR. The FT-IR spectra (Figure 3) showed different peaks at 676.87 functional group is alkynes, 853.86 functional group is aromatic, 1002.31 functional group is alkenes, 1240.54 functional group is amine, 1405.68 functional group is sulfonyl chloride, 1456.14 functional group is Alkane methyl group, 1742.65 functional group is aldehyde, 2359.71 functional group is carbon dioxide, 2980.89 functional group is amine salt, 3282.04 functional group is alcohol (Table 2).

**TABLE 2:** Fourier Transform Infrared Analysis (FTIR) analysis of *Padina antillarum.*

IR frequency range (cm^–1^)	IR frequency (cm^–1^)	Functional group (stretch)	Compound class
3550–3200	3282.04	O-H	Alcohol
3000–2800	2980.89	N-H	Amine salt
2349	2359.71	O = C = O	Carbon dioxide
1740–1720	1742.65	C = O	Aldehyde
1465–1375	1456.14	C-H	Alkane methyl group
1410–1380	1405.68	S = O	Sulfonyl chloride
1250–1020	1240.54	C-N	Amine
1000–650	1002.31	=C-H	Alkenes
900–675	853.86	C-H	Aromatics
700–610	676.87	-C = C-H:C-H	Alkynes

### Isolation of Lipids

**FIGURE 4:**
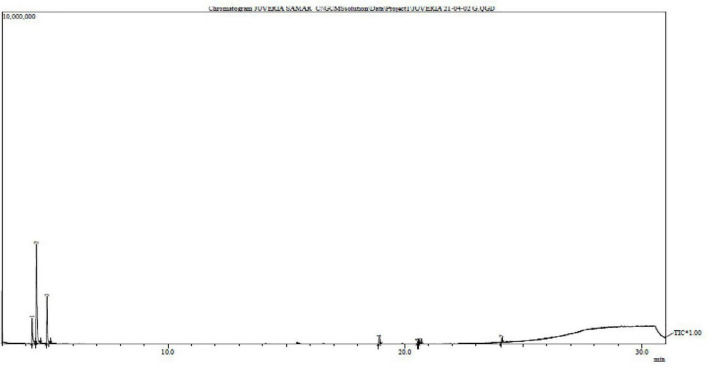
Gas chromatography – mass spectrometry chromatogram of *Padina antillarum* (Kützing) Piccone.

Approximately, 257 g of dry seaweed powder was macerated in the methanol and 20.12 g of crude extract was obtained. According to Ghazala et al. (2004a,b) esterification process was performed and run the column chromatography. The fractions obtained from the column chromatography were directly injected into the GC-MS. The GC-MS of Lipids (Figure 4). Some of the prominent radicals are given in Table 3.

**TABLE 3:** Phycochemical analysis *Padina antillarum* by GC MS.

Compound name	Molecular formula	Molecular weight	Relative %age
Tetradecane	C_14_H_30_	198	3.04
Penta decone	C_15_H_32_	212	1.53
Diethyl Phthalate	C_12_H_14_O_4_	222	2.02
Hexadecane	C_16_H_34_	226	3.55
Phthalic acid	C_13_H_14_O_4_	234	1.53
Oxirane, tetradecyl	C_16_H_32_O	240	6.12
Detadecane	C_18_H_38_	254	1.53
Didodecyl phthalate	C_32_H_54_O_4_	502	4.08
12 Hexadecadirnoic acid	C_17_H_30_O_2_	266	3.57
Methyl palmitoleniate	C_17_ H_32_O_2_	268	20.8
Palmitic acid	C_17_H_34_O_2_	270	2.02
1,2-Benzenedicarboxylic acid	C_16_H_22_O_4_	278	3.57
Hexadecanoic acid	C_18_H_36_O_2_	284	4.08
9, 12 Octadecadicnoic acid	C_19_H_34_O_2_	294	1.51
9 Octadecenoic acid (Oleic acid)	C_19_H_36_O_2_	296	3.57
Octadecanoic acid	C_19_H_38_O_2_	298	2.08
Nonadecenoic acid	C_20_H_38_O_2_	310	5.10
Docosenoic acid	C_23_H_44_O_2_	352	6.57
1,2-Benzenedicarboxylic acid diisooctyl esta	C_24_H_38_O_4_	390	4.59
Didodecyl phthalate	C_32_H_54_O_4_	502	4.08
1,2-Benzene dicarboxylic acid, ditricdecyl ester	C_34_H_54_O_4_	530	3.59
Tetrasiloxane	C_16_H_50_O_7_Si_8_	578	0.59
Cyclononasiloxane	C_18_H_54_O_9_Si_9_	666	6.11
Cyclode casiloyane cicosamethyl	C_20_H_60_O_10_Si_10_	740	4.10

### Biological Activities

#### Antioxidant Activity of *Padina antillarum* Extracts

##### 2,2′Azino-Bis (3–Ethylbenzothia Zoline–*b*-Sulfuric Acid) Assay

**FIGURE 5:**
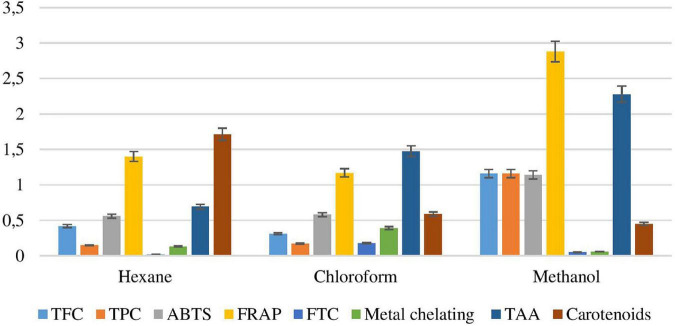
Antioxidant activity of *Padina antillarum* (Kützing) Piccone.

ABTS^++^ radical cation was produced in the stable form using potassium persulphate. The maximum ABTS^++^ value was 600.47 from the methanol extract while the minimum value was 293.63 extracted from chloroform extract (Figure 5). The relative antioxidant ability to scavenge the radical ABTS^++^ has been compared with the standard curve Trolox mg/ml.

##### Ferric Reducing Anti-oxidant Power Evaluation

Methanol, *n*-hexane, and chloroform exhibited good FRAP values, i.e., 1985.78, 929.42, and 767.57 TE μM/ml. The crude methanol extract exhibited the highest FRAP value showing a synergic effect of antioxidant constituents while chloroform exhibited the lowest value (Figure 5). Higher FRAP values were obtained for samples in more polar solvents. The highest value of FRAP exhibited the highest level of phenolic and flavonoid contents. The FRAP assay was used to estimate the reduction potential in the solvent. The standard curve Trolox μM/ml.

##### Ferric Thiocyanate Investigation

The Percentage Lipid Peroxidation Inhibition (IP %) of various seaweed extracts (Figure 5) was found between 1216.93 and 1456.6% inhibition of peroxidation. The FTC mg/ml value of seaweeds can also be determined on the basis of butylated hydroxyl toluene (BHT) standard curve. The minimum FTC mg/ml was shown by chloroform extracts at 0.165 mg/ml BHT and the maximum by methanol extract at 8.5 mg/ml BHT.

##### Metal Chelating Activity

The metal chelating potential in terms of % inhibition of ferrozine-Fe^2^ of *P. antillarum* various extracts varied from 48.82 to 92.42%. The minimum potency was indicated by methanol extract and the maximum by chloroform extract (Figure 5).

##### Total Anti-oxidant Activity

The total antioxidant activity of different crude extracts of *P. antillarum* was investigated by Phosphomolybdenum assay (Figure 5). The maximum antioxidant potential was exhibited by methanol extract, i.e., 911.88 mg/ml of ascorbic acid, while the minimum effectiveness was shown by chloroform, i.e., 277.88 mg/ml ascorbic acid. The total antioxidant value was calculated according to the ascorbic acid standard curve.

##### Total Carotenoid Content

The carotenoid concentration in extracts of *P. antillarum* ranged between 45.24 and 326 β-cE mg/100 g (β-carotene Equivalent mg per 100 g). The least contents were observed in methanol macerates in addition to higher concentrations reported in *n*-hexane extracts (Figure 5). Results were calculated with reference to the β-carotene standard curve.

##### Total Flavonoid Content

Flavonoids are responsible for many biological activities such as anti-cancer, antimicrobial, antiviral, anti-inflammatory. The antimicrobial activity of flavonoids is commonly used in Chinese traditional medicine. Adjuvant arthritis has been cured by Rutin (Guardia et al., 2001). The flavonoids from *P. antillarum* were found in methanol extract 11.07 while the minimum value in chloroform 2.935 (Figure 5). The values were referred to in the standard curve of Rutin as RE μg/g.

##### Total Phenolic Content

A major source of bioactive compounds is seaweeds. Approximately 850 phenolic compounds are known, mostly belonging to marine algae. The maximum amount of phenolics was extracted from methanol 235.68 mg/ml GAE while the minimum was from *n*-hexane 20.78 mg/ml GAE extract of *P. antillarum* (Figure 5). The values were calculated in comparison with the gallic acid standard curve and expressed in terms of GAE mg/ml

##### 2,2 – Dipheyny-1-Picrylhydrazyl Radical Scavenging Assay

**FIGURE 6:**
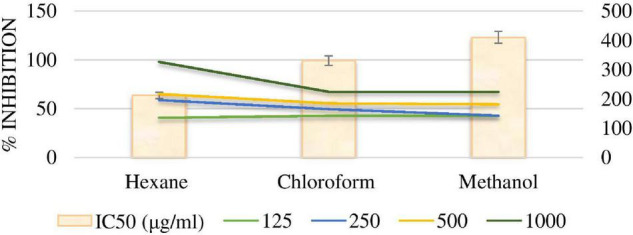
%DPPH & IC _50_ free radical scavenging activity of extracts.

DPPH potential obtained by different fractions was compared with the standard Ascorbic acid curve (Figure 6). The methanol extract of the *P. antillarum* yields a maximum value (84.97) at a concentration of 1,000 μl. The DPPH potential ranged from 50.99 to 84.97% at various concentrations of different extracts. The DPPH percentage was dependent on the concentration of the sample, i.e., more the concentration more the % scavenging. Inhibition concentration 50% of the sample was determined to check the greater antioxidant potential and vice versa. The methanol extract showed the least IC_50_ value, i.e., 1.173 μg/ml followed by *n*-hexane 10.89 μg/ml and chloroform 40.66 μg/ml (Figure 6).

#### Antimicrobial Activity

##### Antibacterial Activity of Padina antillarum Extracts

**FIGURE 7:**
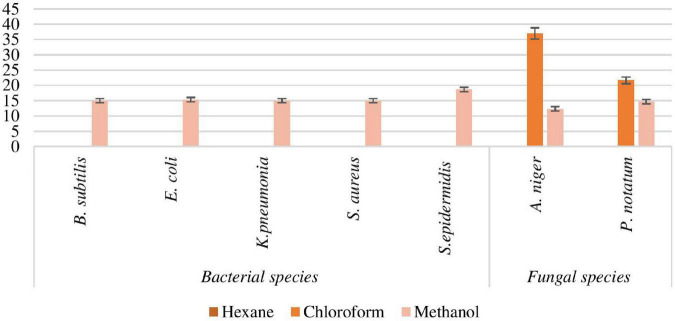
Antimicrobial activity of *Padina antillarum* (Kützing) Piccone.

A number of seaweed species have been already reported for antimicrobial activity. The zone of inhibition of selected seaweed extracts is shown in Figure 7. The methanol extract exhibits the maximum zone against *Staphylococcus epidermidis* (18.66 ± 0.234 mm) while the *n*-hexane extract inhibits the zone against *Bacillus subtilis, Escherichia coli*, and *Klebsiella pneumonia*. There was no zone formation of *n*-hexane extract against *B. subtilis, E. coli*, and *K. pneumonia* while no zone formed of chloroform extract against *B. subtilis, E. coli, S. aureus*, and *S. epidermidis*. The zone of inhibition of methanol extract was 15.00 ± 0.087, 15.33 ± 0.28, 15 ± 0.0045, 16.09 ± 0.0032, and 18.66 ± 0.09 against *B. subtilis, K. pneumonia, E. coli, S. aureus*, and *S. epidermidis*, respectively (Table 4). The results of MIC were noticed to have the broadest activity of the extract against the bacteria was 0.125 mg/ml.

**TABLE 4:** Antimicrobial activity of *Padina antillarum.*

Algal species	*n*-Hexane	Chloroform	Methanol
**Antibacterial activity**			
*Bacillus subtilis*	0.00 ± 0.00	0.00 ± 0.00	15 ± 0.087^a^
*Escherichia coli*	0.00 ± 0.00	0.00 ± 0.00	15.33 ± 0.28^a^
*Klebsiella pneumonia*	0.00 ± 0.00	0.00 ± 0.00	15 ± 0.0045^a^
*Staphylococcus aureus*	0.00 ± 0.00	0.00 ± 0.00	16.09 ± 0.003^ab^
*Staphylococcus epidermidis*	0.00 ± 0.00	0.00 ± 0.00	18.66 ± 0.09^c^
**Antifungal activity**			
*Aspergillus niger*	0.00 ± 0.00	37 ± 0.012^b^	12.33 ± 0.004^a^
*Penicillium notatum*	0.00 ± 0.00	21.66 ± 0.03^a^	18 ± 0.03^b^

*Data presented as mean of triplicates ± indicates standard error. Mean followed by different letters in the same column are significantly different at p < 0.05 according to Duncan’s multiple range test.*

##### Antifungal Activity of Padina antillarum Extracts

The chloroform extract inhibits the maximum zone against *Aspergillus niger* (37.00 ± 0.012 mm) and methanol extract showed 12.33 ± 0.004 mm while the *n*-hexane extract gives no zone against *A. niger* (Figure 7). The zone of inhibition of chloroform and methanol extract was 21.66 ± 0.03, 18.66 ± 0.43 against *Penicillium notatum*, respectively.

##### Anthelmintic Activity of Padina antillarum Extracts

**FIGURE 8:**
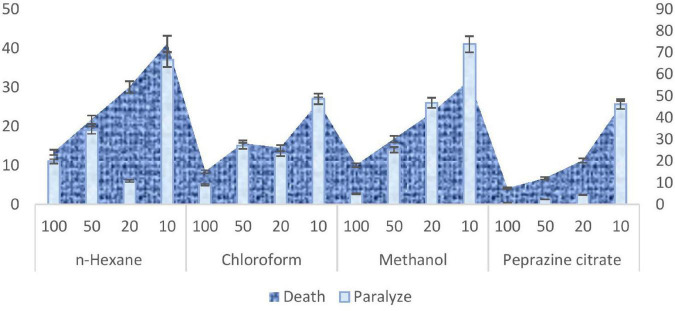
Anthelmintic activity of *Padina antillarum* (Kützing) Piccone.

Helminthes are the main disease causing in the human being and animals. The treatments available in market never remained up to the mark or have developed resistance causing recurrent attacks. For this purpose, the anthelmintic activity was taken into consideration. The anthelmintic potential of various extracts of *P. antillarum* was evaluated at four concentrations (10, 20, 50, and 100 μg/ml). In 4 h treatment period, the paralysis and death time was calculated for various working concentrations. The paralysis time duration in *P. antillarum* extracts ranged from 1.1 to 4.1 min. The maximum time was taken by *n*-hexane extracts indicating its least anthelmintic activity. The most potent results were shown by methanol extracts which paralyzed the *Haemonchus contortus* within 1 min. The overall examination of various concentrations indicated that methanol extract had the highest potential followed by chloroform and hexane. The *n*-hexane extract was documented as the least efficient. The treatment period for death varied between 1.5 and 7.4 min at various concentrations (Figure 8). The shortest death period was shown by methanol extract while the highest time duration was taken using *n*-hexane extract.

##### Antidiabetic Activity of Padina antillarum Extracts

Antidiabetic activity of P. antillarum extracts is divided into two phases.

##### Toxicity Test

The methanolic extracts of *P. antillarum* were observed to be non-significant which did not produce any change in autonomic or behavioral responses during the study. No mortality was observed up to the 7th day of monitoring in any group of mice.

##### Antidiabetic Test

**FIGURE 9:**
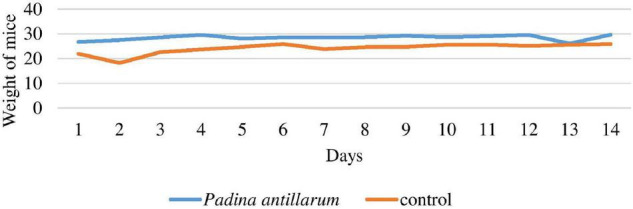
Weight of mice with reference to control group.

**FIGURE 10:**
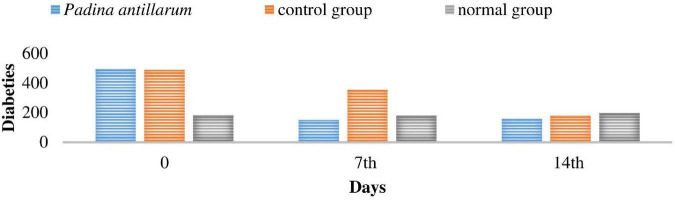
The blood glucose level of the mices during the study.

The mean body weight of mice was significantly *p* < 0.05 increased as compared to the normal mice (Figure 9). The blood glucose level was also checked on fasted mice and compared the values with control mice. The blood glucose level of each mouse was checked prior to the Alloxan administration the basal level of plasma glucose was not significantly higher than 300 in the mice selected for the study. The blood glucose level was checked on 0, 7, and 14 days of the study. *P. antillarum* extract decreases the blood glucose level as compared to other groups (Figure 10).

##### Hematological Parameters

On the 14th-day, a complete blood screening of the mice have done. Table 5 shows the hematological parameters of the study period *P. antillarum.* This extract has a high Hb level as compared to Glipizide, with positive control and negative control. RBC, WBC, Hb, and platelet count showed a significant increase was seen in the mean count (Table 6). In diabetes, elevated levels of serum urea and creatinine are observed which may be due to renal damage caused by abnormal glucose regulation. In the present study, a significant increase in serum urea and creatinine levels were observed in diabetic mice compared to normal control mice. The treatment with *P. antillarum* methanolic extract lower the above parameters compared to diabetic control mice and it showed a protective effect on kidney. Under diabetic conditions, the occurrence of reduction of protein and albumin may be due to increased protein catabolism which is clinical markers. After the treatment with *P. antillarum* extract, the albumin and protein levels were seen to be increased.

**TABLE 5:** Effect of *Padina antillarum* extract on hematological parameters.

Test	Reference value	*Pad. antillarum*	Glib	Positive control	Negative control
**Liver function test**					
Bilirubin Total (mg/dL)	>0.1	0.2 ± 0.000	0.2 ± 0.000	0.225 ± 0.000	0.2 ± 0.004
A.L.T (S.G.P.T) (U/L)	6–19	179 ± 0.012	17.5 ± 0.009	83.75 ± 0.018	46.5 ± 0.008
A.S.T (S.G.O.T) (U/L	167–513	409 ± 0.150	82 ± 0.013	317 ± 0.145	274 ± 0.167
Alkaline Phosphatase (U/L)	93–387	9 ± 0.003	211 ± 0.198	9 ± 0.005	9 ± 0.008
Protein Total (g/dL)	6.4–7	4.9 ± 0.002	–	6.82 ± 0.002	8.35 ± 0.003
Albumin (g/dL)	2.7–3.9	1 ± 0.005	–	1.2 ± 0.001	1.6 ± 0.003
A/G Ratio	1.2–2.2	0.3 ± 0.000	–	0.2 ± 0.000	0.2 ± 0.000
**Renal function test**					
Serum Creatinine (mg/dL)	0.2–0.5	0.05 ± 0.002	0.4 ± 0.000	0.1 ± 0.001	0.1 ± 0.001
Serum Urea (mg/dL)	12.3–24.6	125 ± 0.006	32 ± 0.003	50 ± 0.008	52 ± 0.007
**Lipid profile**					
Serum Total Cholesterol (mg/dL)		86 ± 0.009	71.3 ± 0.016	143.5 ± 0.100	141.5 ± 0.140
Serum Triglycerides (mg/dL)	1–10	41 ± 0.004	14.1 ± 0.002	167.2 ± 0.129	297.5 ± 0.106
Serum HDL – Cholesterol (mg/dL)		34 ± 0.009	57.2 ± 0.007	62 ± 0.004	50 ± 0.009
Serum LDL – Cholesterol		19 ± 0.001	21.4 ± 0.004	26 ± 0.002	33.5 ± 0.007
Serum Non – HDL Cholesterol		52 ± 0.005	57.8 ± 0.007	81.5 ± 0.007	91.5 ± 0.008

**TABLE 6:** Complete blood test of the mice.

Test	Reference value	*Pad. antillarum*	Glib.	Positive control	Negative control	Water
HB (g/dL)	8–12	13 ± 0.005	5.8 ± 0.001	8.63 ± 0.001	11 ± 0.000	7.2 ± 0.004
RBC Count (1012/L)	8–18	7.6 ± 0.001	3.0 ± 0.001	7.61 ± 0.001	7.5 ±	4.8 ± 0.005
HCT (%)	22–38	42 ± 0.002	15.9 ± 0.002	35 ± 0.003	39 ± 0.005	23 ± 0.007
MCV (fL)	16–25	55 ± 0.001	49 ± 0.002	53.2 ± 0.004	52 ± 0.009	49 ± 0.003
MCH (pg)	5.2–8	17 ± 0.002	30 ± 0.002	14.5 ± 0.005	15 ± 0.008	14 ± 0.006
MCHC (%)	30–36	31 ± 0.003	63 ± 0.003	27.5 ± 0.002	29 ± 0.008	30 ± 0.002
WBC Count (109/L)	4–13	4.2 ± 0.001	9.45 ± 0.001	3.05 ± 0.001	4.09 ± 0.002	1.02 ± 0.000
Neutrophils (%)	30 – 48	44 ± 0.002	29 ± 0.002	18.7 ± 0.002	20 ± 0.004	29 ± 0.003
Lymphocytes (%)	50–70	7.9 ± 0.001	36.15 ± 0.001	52.3 ± 0.002	51 ± 0.002	16.8 ± 0.004
Monocytes (%)	>4	47 ± 0.002	28.8 ± 0.003	24.2 ± 0.003	23 ±	53 ±
Eosinophils (%)	1–8	0.4 ± 0.000	0.45 ± 0.000	2.4 ± 0.001	1.9 ± 0.001	0.4 ± 0.000
Platelet Count (^109^/_L_)	300–600	1129 ± 0.002	372.5 ± 0.004	685 ± 0.003	845 ± 0.001	55 ± 0.001

##### Oral Glucose Test

**FIGURE 11:**
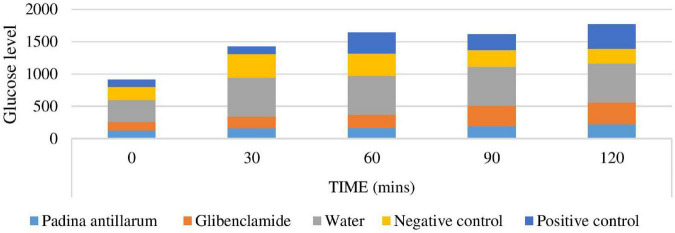
Oral glucose test of the mice.

Oral glucose test is used extensively for the diagnosis of type 2 diabetes. This test displays the competence of the diabetic individual to effectively utilize glucose after a meal. The oral glucose test resembles the glucose and insulin dynamics of physiological conditions closely (Figure 11). Results of the oral glucose test revealed that *P. antillarum* methanolic extract curve was significantly *p* < 0.05 attenuated at both low and high doses.

## Discussion

Algal specimens were collected in drifted conditions from various sites (Buleji, Haji Ghot, Chasma Ghot, Ali Ghot French beach, Hawks bay, and Manora) on the Karachi coast. All these coasts have a lavish amount of sea weed growth at the intertidal zone of the sea, which comes out mostly and collected as drifted conditions as well as scratched from the exposed rocks during the low tides (Khan, 2017). The different extracts of *P. antillarum* revealed the presence of alkaloids, terpenoids, saponins, tannins, steroids, and phenols in phycochemical tests. All these secondary metabolites have been extensively used in the pharmaceutical industry (Ravi et al., 2017). The ash, moisture, and porosity of the sample are similar to the recent publication of Anis et al. (2018). Ash content of *P. antillarum* powder was high if compared with *P. australis* only 34.58%. It is also different from other species such as *P. minor* 30.53% (Felix and Brindo, 2014). Ash contents in a material could be related to a number of mineral components (Ratana-arporn and Chirapart, 2006). The mineral components of macroalgae could be affected by the processing method as well (Ruperez, 2002). Tannins bind to proline-rich protein and interfere with protein synthesis. Tannins have anthelmintic, antioxidant properties and are used in remedies as an antimicrobial and antiviral agent. Terpenoids have antioxidant activity for cancer treatment. Antimicrobial, cytotoxic, and antiplasmodic properties are commonly found in alkaloids (Nugraha et al., 2019). Steroids isolated from marine algae have medicinal value, saponins have unique residues like 2,3-dihydro-2, 5-dihydroxy-6-methyl-4H-pyran-4-one (DDMP) which allows saponins to scavenge superoxides by forming hydroperoxide intermediates which prevent bio-molecular damage (Kumar et al., 2015). Even though the bioactive compounds have different contents, all the active compounds of *Padina* species can be used for pharmaceutical properties such as inhibiting the growth of the pathogenic organisms (Salosso et al., 2020). The highest mineral contents of *P. antillarum* were calcium 258.943 followed by sodium 17.6543 and the lowest was cadmium-0.0279. Other minerals such as potassium 14.972, magnesium 10.7506, iron 4.3142, zinc 0.3940, and lead 0.0151. A similar result is also reported by Manteu et al. (2018) which has the highest mineral contents of *P. minor* calcium (32.91 mg/g) and potassium (26.9 mg/ml). Although the highest mineral content is found in the same mineral in three *Padina* species. It means that the mineral contents of the *Padina* species are affected by species and habitat (Salosso et al., 2020).

Fourier Transform Infrared Analysis spectra were used to identify the active components based on the peak value in the region of infrared radiation. The *P. antillarum* spectra absorption peaks near the Ponnanikajamideen et al. (2014) results on *P. tetrastromatica.* The peaks were identified as alcohols, phenols, secondary amine, halide group, and nitriles. An increasing number of investigations of volatile compounds from non-essentials to essential oils from marine algae have been published in recent years (Kamenarska et al., 2002). The volatile compounds were investigated by GC/MS and the result is summarized in Table 3. Most of the fatty acids are identified as common for marine organisms and are normally found as esters in different groups of lipids. The main part of the polar compound as free fatty acids. Their composition was typical of the genus with palmitic acid being the main fatty acid followed by oleic acid similar to Kamenarska et al. (2002) findings. Palmitic acid appears to be the most abundant fatty acid in brown algae (Aknin et al., 1992).

Seaweeds produce varied and versatile biomass useful for multiple applications. They can be used in a broad variety of formats (e.g., fresh, dried, powder or flakes, salted, canned, liquid extracts or as prepared foods) for direct human consumption or processed into food additives and nutraceuticals, feeds, fertilizers, biofuels, cosmetics and medicines, amongst others (Anis et al., 2017).

Algae produce bioactive compounds with rich pharmacological potential. They generate these compounds as a response to environmental conditions or characteristics (competition for space, maintenance of unfolded surfaces, repulsion of predators, etc.). According to a recent article (Chingizova et al., 2017) isolated chemical compounds from marine seaweed have been shown to owe bioactivities such as antimicrobial, antioxidant, and anti-inflammatory properties, as well as anticoagulant and apoptotic effects.

Flavonoids are proved to have antitumor and antioxidant properties (Kopustinskiene et al., 2020). Flavonoids were present in all samples. These are the major disease fighters on human health. Phenolic compounds have specific physical, chemical, and biological properties which make them a superior drug constituent against disease. These are the main fighter for antimicrobial, anti-inflammatory, antiviral, and anticancer diseases. Phenolics, flavonoids, and carotenoid contents may be varied according to the extraction solvents. The phenolic compounds that have been isolated from seaweeds are scarce, and further research will enlarge the biochemical library and improve the chance to discover new potential compounds for different industries or areas, so this area is still evolving along the road from isolation to application. The major problem of these compounds to be inserted in real commercial applications is mainly the compound concentration in seaweed. Most of the seaweed phenolic pharmaceutical and biomedical bioavailability studies have been supported in mouse-model systems. Phenolic compounds are the most researched seaweed compounds and are already applied in commercial solutions, e.g., cosmetic products (Cotas et al., 2020). The highest phenolic contents were observed in *P. antillarum* methanolic extract. These findings are similar to the finding of Sameeh et al. (2016) as *Padina* extract has higher phenolic values among the *Enteromorpha.*

Antioxidants are considered to be effective protecting tools against oxygen species and their derivatives, produce inside living cells during biochemical processes. Specifically, natural antioxidants are very important for the last three decades because of health issues with synthetic antioxidants available on the market. Natural antioxidants can be extracted from terrestrial as well as aquatic sources, e.g., phenolics, terpenoids, and flavonoids (Yan et al., 1999). Antioxidant compounds play an important role to prevent health from harmful factors. It is known that seaweeds contain several bioactive compounds with potential/higher antioxidant activity as compared to the terrestrial plants due to the presence of up to eight interconnected polyphenols rings (O’sullivan et al., 2011). Antioxidant activity of seaweeds is due to the presence of pigments chlorophylls, xanthophylls (fucoxanthin), carotenoids, vitamins (vitamins B^1^, B^3^, C, and E), and vitamin precursors such as α-tocopherol, β-carotene, lutein, and zeaxanthin, phenolics such as polyphenols (gentisic acid, phloroglucinol, gallic acid, protocatechuic acid), flavonoids (i.e., rutin, quercetin, myricetin, flavones, flavonols, flavanones, chalcones, hesperidin and flavan-3-ols, isoflavones, methylated flavones), lignins, tocopherols, tannins, and phenolic acids and hydroquinones, phospholipids, particularly phosphatidylcholine, terpenoids, peptides, and other antioxidative substances, which directly or indirectly contribute to the inhibition or suppression of oxidation processes (Farvin and Jacobsen, 2013). The FRAP, % inhibition, and total antioxidant activity value of *P. antillarum* methanolic extract were the highest similar to the results of Chia et al. (2015). An excessive amount of reactive oxygen species may result in lipid peroxidation, which changes the structure of body biomolecules, causing cellular disorders, premature aging, mutations, or cell death. Different researches have demonstrated seaweed antioxidant capacity *in vitro*, attributed to the presence of new antioxidant compounds like carotenoids, certain polysaccharides, and polyphenols, which show scavenger activity, being able to neutralize those reactive oxygen species through their own oxidation, since their affinity to those oxidative compounds is very high (Vieira et al., 2018).

The antimicrobial activity of seaweeds may be influenced by some factors such as the habitat and the season of algal collection, different growth stages of algae, experimental methods, *etc*. Although a variety of solvents have been employed in screening seaweeds for antimicrobial activity (Manivannan et al., 2011). The potential of seaweed as a source of compounds active against pathogenic microorganisms has been confirmed in different studies. Padmakumar and Ayyakkannu (1997) screened 80 species against bacterial and fungal pathogens. Of the algae, 70% exhibited antibacterial activity but only 27.5% showed antifungal activity (Pérez et al., 2016). Rajasekar et al. (2019) have reported that seaweeds are an excellent source of components such as polysaccharides, tannins, flavonoids, phenolic acids, bromophenols, and carotenoids has exhibits different biological activities. Depending upon their solubility and polarity, different solvents show different antimicrobial activity. According to Rajasekar et al. (2019), *P. gymonospora* exhibit maximum activity to *B. cereus* and minimum activity against *C. albicans, D. delicatula* showed better activity toward *P. aeruginosa* and lower activity against *S. aureus.* In the current study *Padina antillarum n*-hexane, chloroform extract showed no zone of inhibition against *B. subtilis, E. coli, K. pneumonia, S. aureus*, and *S. epidermidis.* The methanolic extract of *P. antillarum* exhibits a maximum zone of inhibition against *S. epidermidis* (18.66 ± 0.09) similar to the findings of Madkour et al. (2019).

Seaweeds have been recognized as potential sources of antibiotic substances. The results of the study clearly reveal that the higher the concentration the extract became faster due to the paralytic effect and shorter due to the death. The anthelmintic activity of all the extract has a wide range of chemical classes as per the result of GC-MS. The anthelmintic results can be compared to the Selvi and Santhanam (2016) findings. All the anthelmintic findings compare with the standard drug piperazine citrate available on the market. The results showed dose-dependent anthelmintic activity. It was found that higher concentrations of the extract became faster due to the paralytic effect and shorter due to the death time of all the helminthes, respectively. The current investigation leads to the conclusion that all the subjected seaweed extract have potent anthelmintic activity which may be due to the phytochemicals compounds in the seaweed.

Seaweeds are rich in dietary fibers, unsaturated fatty acids, and polyphenolic compounds. Many of these seaweed compositions have been reported to be beneficial to human health including in managing diabetes. Polysaccharides and dietary fibers from seaweed may improve post-prandial satiety feeling, thereby they assist in the reduction of blood glucose levels and improved insulin sensitivity. Seaweed dietary fibers are also helpful in reducing body weight or weight maintenance; hence they are beneficial in attenuating the risk of obesity (Sharifuddin et al., 2015). Diabetes mellitus is one of the most familiar chronic diseases associated with carbohydrate metabolism. It is also an indication of co-morbidities such as obesity, hypertension, and hyperlipidemia which are metabolic complications of both clinical and experimental diabetes. Despite the fact that diabetes mellitus has a high prevalence, morbidity, and mortality globally; it is regarded as a non-curable but controllable disease. Different synthetic drugs, remedies, and dietary modification play an efficient role in the reduction of the suffering that it causes (Balamurugan et al., 2014). According to Radhika and Priya (2015), fucoxanthin which was found in the brown seaweed may inhibit the accumulation of fats causing obesity and can be used as an antidiabetic drug. In general, increased hepatic glucose production plus decreased hepatic glycogen synthesis and glycolysis are the major symptoms of type II diabetes that results in hyperglycemia. In the current study diabetic condition elevated blood glucose reduced the body weight but by the seaweed extract treatments, the loss of weight is less which is similar to the Balamurugan et al. (2014). A diabetic patient has specific type of blood lipid profile by elevated serum, triglycerides, total cholesterol, and low HDL cholesterol. The mice treated with *P. antillarum* methanolic extract resulted in a low level of triglycerides, total cholesterol, and elevated HDL cholesterol level.

## Conclusion

Marine alga *P. antillarum* is an important resource and industrial applications of these algae drive continuous research to reveal new findings notably for commercial interest as well as versatile sustainable ecological development. The extracts of *P. antillarum* are an important rich source for the development of compounds with medicinal properties. The algal extracts comprising macronutrients, ashes, carotenoids, carbohydrates, lipids, and protein composition were also recorded. The present studies related to algal extracts are important as they can serve as new drug therapeutics with novel activity, as well as a possible solution for the treatment of diseases like diabetes. It is important to develop a strategy to use local algae resources.

The finding of this study suggests that *P. antillarum* extracts *have* a treasured phycochemistry and high antioxidant activity. The antimicrobial activity of the methanolic extract is good as compared to others while the use of this seaweed may minimize the complication of diabetes II (more insulin production in the body) but further studies are necessary to corroborate these results for the dietary recommendation for patients.

### Future Perspective

*Padina antillarum* extract will be used as a dietary supplement due to its high nutritional value. It is economical to use *P. antillarum* extracts for the isolation of compounds that are used in biofuel, dying, pharmaceutical, and cosmetic industries. The high antioxidant value of seaweed may lead to the treatment of different diseases like cardiovascular, diabetes, and cancer treatment.

## Data Availability Statement

The original contributions presented in this study are included in the article/supplementary material, further inquiries can be directed to the corresponding author/s.

## Author Contributions

JS performed and conducted the experiments. GB supervised the manuscript and contributed the research design and validation. AAS contributed to the review, writing, and drafting the manuscript. ANS contributed to the review design of research and funding. SA drafted and funded the manuscript. BLJ guided the statistical analysis NRA shaped the revised manuscript. MH helped with the analysis of experimental results and funding. All authors contributed to the article and approved the submitted version.

## Conflict of Interest

The authors declare that the research was conducted in the absence of any commercial or financial relationships that could be construed as a potential conflict of interest.

## Publisher’s Note

All claims expressed in this article are solely those of the authors and do not necessarily represent those of their affiliated organizations, or those of the publisher, the editors and the reviewers. Any product that may be evaluated in this article, or claim that may be made by its manufacturer, is not guaranteed or endorsed by the publisher.
